# Effects of P-MAPA Immunomodulator on Toll-Like Receptors and p53: Potential Therapeutic Strategies for Infectious Diseases and Cancer

**DOI:** 10.1186/1750-9378-7-14

**Published:** 2012-06-18

**Authors:** Wagner J Fávaro, Odilon S Nunes, Fabio RF Seiva, Iseu S Nunes, Lisa K Woolhiser, Nelson Durán, Anne J Lenaerts

**Affiliations:** 1 Department of Structural and Functional Biology, Institute of Biology, University of Campinas (UNICAMP), CP-6109, 13083-865, Campinas, SP, Brazil; 2 Farmabrasilis R&D Division, Campinas, SP, Brazil; 3 Department of Microbiology, Immunology, and Pathology, Colorado State University, Fort Collins, Colorado, USA; 4 Institute of Chemistry, Biological Chemistry Laboratory, UNICAMP - University of Campinas, Campinas, SP, Brazil; 5 Center of Natural and Human Sciences, Universidade Federal do ABC, Santo André, SP, Brazil

**Keywords:** Bacillus Calmette-Guerin, Immunotherapy, Toll-like receptor, p53, Infectious diseases, *Mycobacterium tuberculosis*, Bladder cancer.

## Abstract

**Background:**

Compounds that can act as agonists for toll-like receptors (TLRs) may be promising candidates for the development of drugs against infectious diseases and cancer. The present study aimed to characterize the immunomodulatory effects of P-MAPA on TLRs in vitro and in vivo, as well as to investigate its potential as adjuvant therapy in infectious diseases and cancer.

**Methods:**

For these purposes, the activity of P-MAPA on TLRs was assayed in vitro through NF-κB activation in HEK293 cells expressing a given TLR, and using an in vivo animal model for bladder cancer (BC). The antimicrobial activity of P-MAPA was tested against *Mycobacterium tuberculosis* (TB) in vitro in an MIC assay, and in vivo using an aerosol infection model of murine tuberculosis. Antitumor effects of P-MAPA were tested in an animal model with experimentally induced BC. Moxifloxacin (MXF) and Bacillus Calmette-Guerin (BCG) were used as positive controls in the animal models.

**Results:**

The results showed that P-MAPA, administered alone or in combination with MXF, induced significant responses in vivo against TB. In contrast, the compound did not show antimicrobial activity in vitro. P-MAPA showed a significant stimulatory effect on human TLR2 and TLR4 in vitro. In BC, TLR2, TLR4 and p53 protein levels were significantly higher in the P-MAPA group than in the BCG group. The most common histopathological changes in each group were papillary carcinoma in BC group, low-grade intraepithelial neoplasia in BCG group and simple hyperplasia in P-MAPA group. Concerning the toxicological analysis performed during BC treatment, P-MAPA did not show evidence for hepatotoxicity and nephrotoxicity.

**Conclusions:**

In conclusion, P-MAPA acted as TLR ligand in vitro and improved the immunological status in BC, increasing TLR2 and TLR4 protein levels. P-MAPA immunotherapy was more effective in restoring p53 and TLRs reactivities and showed significantly greater antitumor activity than BCG. The activation of TLRs and p53 may provide a hypothetical mechanism for the therapeutic effects in both cancer and infectious diseases. Taken together data obtained will encourage the further investigation of P-MAPA as a potential candidate for the treatment of cancer and infectious diseases.

## Background

Immunotherapy represents an approach for the treatment of infectious diseases and cancer [1-3]. Effective immunotherapy for chronic infectious diseases or cancer requires the use of appropriate target antigens, the optimization of the interaction between the antigenic peptide, the antigen-presenting cell (APC) and the T cell and the simultaneous blockade of negative regulatory mechanisms that impede immunotherapeutic effects [1,3]. In cancer, besides the impairment of the immunological status associated with the main disease, several therapies can often cause additional immunosuppression creating the conditions for the emergence of infections [1-4]. In this scenario, compounds which can act in the immune system such as new vaccines, vaccine adjuvants and biological response modifiers are considered potential candidates for the treatment of these diseases or conditions. Compounds that target the TLRs may represent starting points for the development of new drugs, since the TLRs are increasingly implicated in the pathogenesis of some diseases [5,6]. In fact, recent advances in TLR-related research have also shown the therapeutic properties of these receptors against several diseases, including infectious diseases and cancer [7-9]. Consequently, compounds that mimic pathogen associated molecular patterns and activate immune cells via TLRs are candidate drugs being developed to treat several diseases and to be used as vaccine adjuvants. TLRs are transmembrane proteins that recognize pathogen-associated molecular patterns as well as endogenous damage-associated molecular patterns and elicit pathogen-induced and noninfectious inflammatory responses [10,11].TLRs were initially detected only on immune cells, but recent studies demonstrate that tumor cells express functional TLRs and that TLR signaling can promote opposite outcomes: tumor growth and immune evasion or apoptosis and cell cycle arrest [12-14].

Compounds from microbial sources as well as bacterial strains themselves, such as Bacillus Calmette-Guerin (BCG), are used as therapeutic tools in the treatment of some types of cancer, including urothelial cancer [15,16]. BCG is a live, attenuated strain of *Mycobacterium bovis* used widely for tuberculosis (TB) prophylaxis. However, the exact mechanism by which BCG exerts its antitumor effect remains unknown. The treatment of choice for BC is transurethral resection (TUR) and adjuvant therapy with BCG [15-17]. The objective of intravesical BCG therapy is to reduce the risk of recurrence or eradicate carcinoma in situ in patients if complete resection it is not possible [18,19]. BCG administered as a control in the cancer model admittedly acted as TLR2 and TLR4 agonist, since the cell wall of mycobacteria contains certain antigens which are recognized by local macrophages and immature dendritic cells (DC) [20]. Peptidoglycan is an important mycobacterial cell wall component, which is covalently linked to arabinogalactan and mycolic acids [21,22]. In a series of experiments, different research studies have demonstrated that this mycobacterial cell wall component stimulated TLR2 and TLR4 responses in immature DC cells [21,22]. Although intravesical BCG immunotherapy treatment can reduce the risk of recurrence and progression of BC, its use is limited by the adverse effect profile and intolerance that occurs in 20 % of patients, from mild and self-limiting side effects to potentially life-threatening complications, such as systemic BCG infection [23,24].

With respect to infectious diseases, tuberculosis is far from being controlled or eradicated. On the contrary, it is becoming resistant to existing drugs, including most antimicrobial drugs. *Mycobacterium tuberculosis*, the bacterium that causes pulmonary TB remains a major global health problem, infecting nearly one-third of the world’s population and killing at least 3 million people every year [4]. The occurrence of drug-resistant strains of *Mycobacterium tuberculosi*s emphasizes the urgent need for developing new drugs, including those drugs capable of boosting the body’s immune response. Immunotherapy through the use of compounds that act as agonists for TLRs, can represent a valuable approach to infectious diseases and cancer, when used in combination with existing therapies.

P-MAPA can be an immunomodulatory drug candidate. The compound’s ability to fight cancer and infectious diseases was detected in earlier studies using animal models [25-27]. P-MAPA is an acronym for Protein Aggregate Magnesium-Ammonium Phospholinoleate-Palmitoleate Anhydride, a proteinaceous aggregate of ammonium and magnesium phospholinoleate-palmitoleate anhydride, with immunomodulatory properties produced by fermentation of *Aspergillus oryzae*, under development by Farmabrasilis, a non-profit research network [25,28,29]. The compound is a nonlinear biopolymer with molecular mass of 320 kDa. The main components of P-MAPA are Mg^2+^, NH^4+^, phosphate, linoleic acid, palmitoleic acid and protein [25,29-31]. The P-MAPA immunomodulator was originally intended for cancer treatment, and has effectively demonstrated antitumor activity in several animal models including cancer by chemical inducers/promoters [27]. P-MAPA induces immunodulatory effects, including increased cytokine production, mainly interferon-gamma (IFN-γ) and interleukin 2 (IL-2), and stimulates nitric oxide release by macrophages [25,29-31]. These findings expanded the potential therapeutic applications of the compound, suggesting that P-MAPA, like other compounds with immunomodulatory properties, can fight a wide range of infections caused by intracellular pathogens [25,31]. The compound did not show significant toxicity in the preclinical phase when assessed in in vitro cell toxicity assays (V-79 Chinese hamster cell line at concentration of 120 μg/ml) and in vivo acute and chronic toxicity models using Swiss mice (single dose toxicity, oral, at 30 g/kg,), Wistar rats (12-week multiple-dose toxicity, injected subcutaneously, at 1, 10 and 100 mg/kg), and monkeys (*Cebus apella*; 4-week multiple dose toxicity, injected intramuscularly at 5,10 and 30 mg/kg) [28,31]. Moreover, the use of P-MAPA in clinical trial phase I did not show any signs of adverse drug reaction at dosages of 5 mg/square meter, injected intramuscularly, 3 times a week, for 6 weeks [29].

Thus, the present study was aimed to characterize the effects of P-MAPA on TLRs in vitro and in vivo, as well as to assess its potential as adjuvant therapy for infectious diseases and cancer. Regarding its action against cancer, the efficacy of P-MAPA was compared versus BCG in the BC mouse model. And regarding its action against infectious diseases, the in vivo efficacy of P-MAPA was compared alone and co-administered to moxifloxacin (MXF) in a tuberculosis mouse model (Erdman strain).

## Results

### TLR Ligand Screening

**Figure 1:**
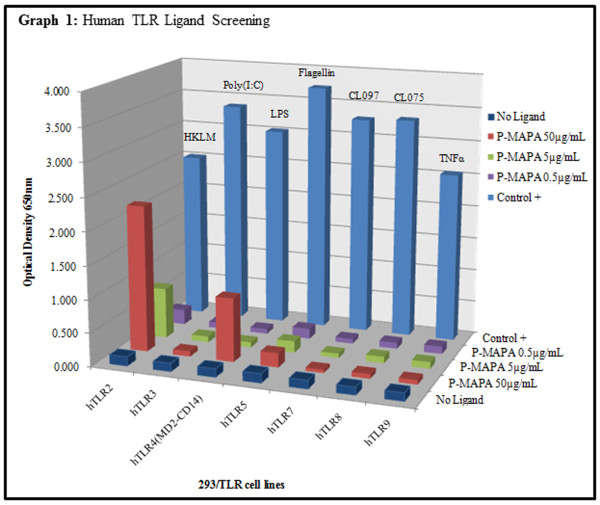
Human TLR ligand screening.

The P-MAPA samples showed a significant stimulatory effect on human TLR2 at a 5 μg/mL concentration corresponding approximately to 30 % of the control ligand (Figure 1). Furthermore, P-MAPA samples showed a significant stimulatory effect on human TLR2 and TLR4 at 50 μg/mL corresponding approximately to 88 % and 32 % of the control ligands, respectively (Figure 1). P-MAPA did not show a significant stimulatory effect on human TLR3, TLR5, TLR7, TLR8 and TLR9 (Figure 1).

### In Vitro Antimycobacterial Activity of P-MAPA

P-MAPA was initially tested using the Rezasurin MIC assay (single point procedure) against H37Rv strain and did not show any antimycobacterial activity in vitro at a concentration of 10 μg/mL (Table 1). Consequently, no further testing of P-MAPA was performed with standard MIC assays (doses range between 0 ug/ml to 0.078 ug/ml) using colorimetric methods. Also, negative and sterility control groups did not show any antimycobacterial activity (Table 1).

**Table 1:** **MICs of Rifampin and Isoniazid as antimicrobial agents and P-MAPA for*Mycobacterium tuberculosis*H37Rv determined by Resazurin MIC Assay**

**Compound**	**Result (μg/mL)**
Negative Control	>10
Sterility Control	>10
P-MAPA	>10
Rifampin	0.006
Isoniazid	0.06

Rifampin (0.006 μg/mL) and isoniazid (0.06 μg/mL) positive controls showed antimycobacterial activity (Table 1).

### In Vivo Antimycobacterial Activity of P-MAPA and MXF

The P-MAPA, MXF and MXF + P-MAPA treatments yielded 0.94, 1.80 and 1.75 log_10_ CFU in lung, respectively, conferring protection to the lung (Table 2). These results showed a statistically significant improvement in the lung (p < 0.001) in comparison with untreated controls (Table 2).

**Table 2:** Plating and Replating of lung and spleen for P-MAPA, MXF and MXF + P-MAPA antimycobacterial activities

**Compounds**	**Tissues**											
	**Lung**				**Spleen**				**Spleen Replate**			
	**Mean CFU**	**log**_**10**_**prot**	**SEM**	**n**	**Mean CFU**	**log**_**10**_** prot**	**SEM**	**n**	**Mean CFU**	**log**_**10**_**prot**	**SEM**	**n**
Untreated Ctrl	4.66	-	0.11	6/6	4.29	-	0.12	6/6	3.34	-	0.18	6/6
MXF	2.86^a^	1.80	0.13	6/6	2.80^a^	1.49	0.23	6/6	1.38^a^	1.96	0.29	5/6
P-MAPA	3.72^a^	0.94	0.10	6/6	3.74	0.55	0.12	6/6	2.31^a^	1.03	0.12	6/6
MXF + P-MAPA	2.91^a^	1.75	0.14	6/6	2.36^a^	1.93	0.32	6/6	0.69^a^	2.65	0.32	4/6

n = Number of mice with CFU/total number of mice at sacrifice; MXF = Moxifloxacin; SEM = standard error of the mean; log_10_ prot = log_10_ CFU of protection; Letter *a*: significantly different from Untreated Ctrl group.

In an initial plating spleen, P-MAPA treatment did not show a statistically significant protective effect on the spleen (p > 0.05) (Table 2). Because of the low bacterial load in the spleens at the initial plating experiment of several animals in the drug treatment groups, the stored undiluted spleen homogenate was re-plated undiluted to increase sensitivity. On replating, the P-MAPA treatment yielded 1.03 log_10_ CFU in the spleen, which showed a statistically significant improvement in spleen status (p < 0.05) when compared to the untreated controls (Table 2).

The combined treatment of MXF + P-MAPA resulted in 1.93 log_10_ CFU of protection in the 1^st^ plating of spleen, which was statistically significant (p < 0.001) compared to untreated controls (Table 2). On replating, the MXF + P-MAPA treatment showed 2.65 log_10_ CFU reduction in the spleen compared to untreated controls (Table 2).

There was no statistically significant difference between MXF administered alone and MXF + P-MAPA (p > 0.05) in either lung or spleen (Table 2). Upon replating of the spleen, there was also no statistically significant difference between MXF administered alone and MXF + P-MAPA (p > 0.05) (Table 2).

### Histopathological Analysis in Bladder Cancer

**Figure 2a - 2e:**
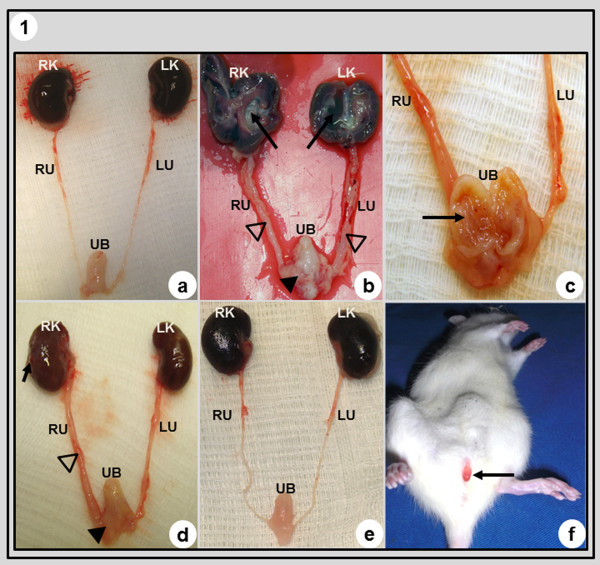
**Histopathological analysis in urinary tract of the animals from CT (a), MNU (b, c), BCG (d) and P-MAPA (e) groups.** In (**a**) and (**e**) the urinary tract showed normal features. (**b**) Lesion widespread in different points of the urinary tract: hydronephrosis the and papillary lesions (arrows) in the kidneys; hydroureter (**open arrowheads**); thickening of the wall the of the urinary bladder and pappillary lesions (**solid arrowhead**). (**c**) Intravesical pappillary lesions (**arrow**); hydroureter. (**d**) Cystic lesions (**arrow**) in the kidney; hydroureter (**open arrowhead**); thickening of the wall of the urinary bladder and papillary lesions (**solid arrowhead**). a - e: **LK** - left kidney, **LU** - left ureter, **RK** - right kidney, **RU** - right ureter, **UB** - urinary bladder. Figure 2**f**: The animals from the MNU and BCG group showed macroscopic haematuria (**arrow**). P-MAPA group showed no macroscopic haematuria.

**Figure 3:**
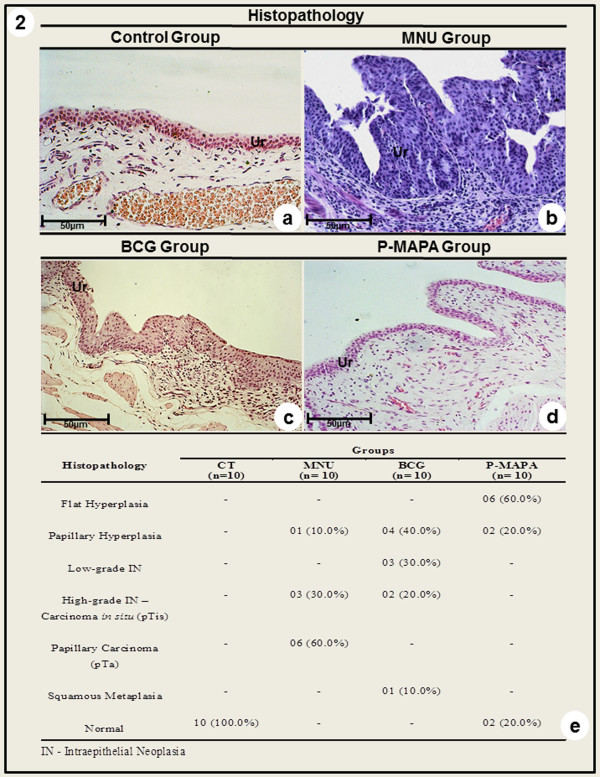
**Photomicrographs of the urinary bladder from Control (a), MNU (b), BCG (c) and P-MAPA (d) groups.** (**a**) Normal urothelium; (**b**) Papillary carcinoma; (**c**) Carcinoma *in situ*; (**d**) Flat hyperplasia. a - d: Ur - urothelium. (**e**) Percentage of histopathological changes of the urinary bladder of rats from CT, MNU, BCG and P-MAPA groups.

The urinary tract in the CT group showed no structural changes (Figures 2a and 3a). However, the urinary tract in the MNU group showed significant histopathological changes such as hydronephrosis and hydroureter (Figures 2b and 2c); papillary carcinoma of the urinary bladder (pTa) and carcinoma in situ (pTis) in 60.0 % and 30.0 % of the animals, respectively (Figures 2b, 2c, 3b and 3e).

Papillary hyperplasia (40.0 %) and low-grade intraepithelial neoplasia (30.0 %) were the more frequent histopathological changes in the urinary bladder in the BCG group (Figures 3c and 3e). Also, the urinary tract in the BCG group showed macroscopic changes such as: cystic lesions in the kidneys, hydroureter, thickening of the wall of the urinary bladder and papillary lesions (Figure 2d).

The macroscopic features of the urinary tract in the P-MAPA group were similar to those found in the CT group (Figure 2e). In the urinary bladder, the more frequent histopathological changes in the P-MAPA group were flat hyperplasia (60.0 %) and papillary hyperplasia (20.0 %) (Figures 3d and 3e).

The occurrence of urinary calculi and macroscopic haematuria was only observed in the MNU and BCG groups, being absent in the P-MAPA group (Figure 2f).

### Western Blotting Analysis of TLR2, TLR4 and p53 in Bladder Cancer

**Figure 4:**
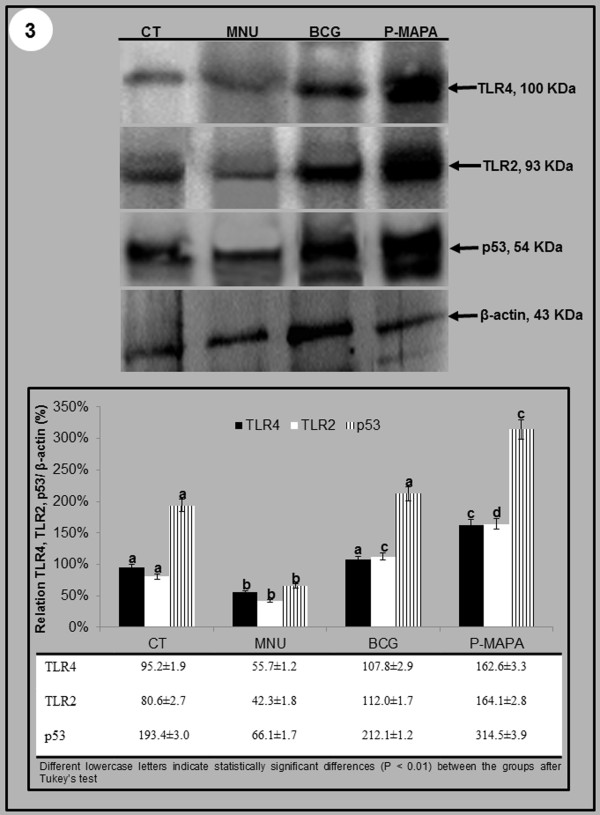
**Representative Western Blotting and semiquantitative determination for TLR2, TLR4 and p53 protiens of the urinary bladder extracts in the four experimental groups.** The protein levels were identified in the blots. β-Actin was used as the endogenous control. Data were expressed as the mean ± standard deviation (n = 5).

The highest TLR4 and TLR2 protein levels were found in the P-MAPA groups compared to the other experimental groups (Figure 4). However, these levels were significantly higher in the BCG group than in the CT and MNU groups (Figure 4).

The p53 protein level was significantly higher in the P-MAPA groups than in the BCG and CT groups. Furthermore, this level was significantly higher in the BCG and CT groups when compared to the MNU group (Figure 4).

### Toxicological Analysis in Bladder Cancer

The ALT and AST levels did not show any statistical difference among the four experimental groups (Table 3). Creatinine and urea levels were significantly higher in the MNU group compared to BCG and P-MAPA groups, which did not show statistical differences (Table 3). The alkaline phosphatase activity was significantly higher in the MNU group compared to the other experimental groups (Table 3).

**Table 3:** Toxicological and Biochemical Biomarkers for Control, MNU, BCG and P-MAPA groups

**Parameters**	**Control**	**MNU**	**BCG**	**P-MAPA**
	**(n = 10)**	**(n = 10)**	**(n = 10)**	**(n = 10)**
ALT (U/L)	49.23 ± 1.61	46.03 ± 0.63	46.30 ± 3.60	51.61 ± 7.07
AST (U/L)	189.72 ± 28.74	133.05 ± 2.29	201.61 ± 23.84	208.38 ± 31.34
Alkaline Phosfatase (U/L)	42.97 ± 0.87^a^	128.11 ± 8.21	38.88 ± 3.51^a^	40.56 ± 3.93^a^
Urea (mg/dL)	55.45 ± 3.90^a^	163.09 ± 14.14	60.52 ± 5.90^a^	57.32 ± 7.16^a^
Creatinine (mg/dL)	1.18 ± 0.26^a^	10.33 ± 0.10	1.22 ± 0.25^a^	1.94 ± 1.24^a^

Data expressed as mean ± SEM, p < 0.05; Letter *a*: significantly different from MNU group. MNU = *n-methyl-n-nitrosourea*; BCG = Bacillus Calmette-Guerin.

## Discussion and conclusions

Compounds that act as agonist for TLRs, or in other words, compounds or molecules that bind to and activate TLRs are the subject of intensive research and development for the treatment of infectious diseases and cancer. In this regard, the present studies detail a series of in vitro and animal models and demonstrate that the immunomodulator P-MAPA exhibits significant antimycobacterial and antitumor activity. TLR activation facilitates and instructs the development of adaptive immune responses by increasing the levels of expression of co-stimulatory molecules such as CD80 and CD86 on DC, allowing DC to more effectively activate T cells [21,32].

An important aspect of the induced cytokine production is the differentiation of T cells either into Th_1_ or Th_2_ subsets, which will guide the pattern of adaptive response launched by the host against the pathogen. For instance, human monocyte-derived DC stimulated with lipopeptide from *Mycobacterium tuberculosis* secretes IL-12 over IL-10, skewing the host's adaptive immune response toward a Th_1_ pattern, characterized by a cellular, cytotoxic T cell response [33].

In TB, recent evidence shows that the TLRs are able to recognize *Mycobacterium tuberculosis*-associated molecular patterns and mediate the secretion of cytokines and other antibacterial effector molecules [34,35]. The immune system recognizes pathogen-associated mole cular patterns (PAMPs) such as the bacteria’s outer cell wall composition of peptidoglycan, mycolic acids, lipomannan, and lipoarabinomannan [36]. PAMPs stimulate immune recognition receptors such as toll-like receptors (TLR) [37,38]. In vitro studies have delineated these mechanisms, providing evidence that TLR2 and TLR4 are stimulated by interactions with *Mycobacterium* cell wall molecules leading to the maturation of DC, macrophages, and production of IFN-γ [39,40]. Other studies suggest that TLRs variants may contribute to human susceptibility to tuberculosis disease [41]. Recent studies in animal models also suggested that TLR, mainly TLR-2 and TLR-4 may play a protective role in host defense against lung infection by *Mycobacterium tuberculosis*[42,43].

The protective immunity against *Mycobacterium tuberculosis* has been ascribed to CD4+ T cell-mediated immunity [42,43]. The breakdown of immune responses designed to contain the infection can result in reactivation and replication of the bacilli, with necrosis and damage to lung tissue [44,45]. An efficient immune response against the intracellular pathogen *Mycobacterium tuberculosis* is critically dependent on rapid detection of the invading pathogen by the innate immune system and the coordinated activation of the adaptive immune response [45]. An effective response against *Mycobacterium tuberculosis* was demonstrated in vivo in the present study. P-MAPA did not show any direct in vitro antimicrobial activity. In vivo P-MAPA alone or combined with MXF did not show synergistic and/or antagonic effects; however P-MAPA induced a significant antimicrobial response against *Mycobacterium tuberculosis.* Future studies are required to investigate whether this result is observed with the use of P-MAPA combined with other TB drugs (such as rifampin or isoniazid), or whether this is a MXF-specific effect. This finding could be relevant for the use of P-MAPA as candidate to be evaluated in antitubercular treatments, with the aim of reducing the doses of antimicrobials or shortening the period required for administration of such drugs.

Ayari et al. [46] have recently shown that TLRs are expressed in normal urothelium and BC. The role of TLRs in cancer is a matter of debate because conflicting data argue that TLRs are negative or positive regulators of cancer [47,48]. Different authors demonstrated that TLRs help tumour cells evade immune system response [12,49] while others showed that TLRs expression on tumour cells might drive them to apoptosis or other types of cell death [50-52]. Activated TLRs on cancer cells may promote cancer progression, invasion, anti-apoptotic activity and resistance to host immune responses [49,53,54]. Some TLRs have been connected with tumor progression and invasion as after TLR stimulation they can increase the expression of matrix metalloproteinases, up regulate different cytokines and chemokines or other inflammatory factors [54-56]. Matijevic *et al.*[57] and Matijevic & Pavelic [58] investigated the difference between primary tumour and metastasis based on TLR3 level and found a huge difference between FaDu and Detroit 562 cell lines, which were of the same origin (pharynx), but FaDu was primary and Detroit 562 metastatic carcinoma. The same authors reported on the dual role of TLR3 in pharynx metastatic cell line (Detroit 562); on the one hand TLR3 activation drove cells to apoptosis, while on the other its stimulation contributed to tumor progression by altering the expression of tumor promoting genes (*PLAURRORB*) and enhancing the cell migration potential. In addition, these authors showed that TLR3 signaling pathway was functional in another metastatic cancer cell line (SW620) suggesting TLR3 might be important in the process of tumor metastasis.

In this context, the administration of TLR agonists was also reported to exert strong antineoplastic effects against established tumors in mice and humans [12,14,59]. TLR activation may also cause tumor regression by increasing vascular permeability and through the recruitment of leukocytes, which determines tumor cell lysis by *natural killer* (NK) and cytotoxic T cells [59]. Accordingly, one of the most promising effects of TLR stimulation by specific agonists in cancer therapy is the activation of the adaptive immune system [14,60].

The contradictory evidence that TLR promotes carcinogenesis, whereas in others it exerts antitumor effects, could be explained by the different intensity and nature of the inflammatory response [48]. In fact, chronic inflammatory processes are milder than acute inflammatory responses, which are aimed at inducing pathogen clearance. In most cases, cancer-associated inflammation is similar to chronic inflammation, including the production of factors that stimulate tissue repair and cancer cell survival and proliferation [48]. However, if the inflammatory response develops into acute inflammation, an immune effector mechanism is activated, and cancer regression takes place [48,61]. Among the different elements that control neoplastic processes, a major role is attributed to members of the chemokine superfamily. Chemokines expressed by tumor cells and by host cells play a critical role in determining the fate of the developing tumor by regulating the migration of different leukocyte subtypes [48,62]. The relative proportion of each defense cell type (macrophages, T cells, NK cells, dendritic cells, or other leukocyte subtypes) within the tumor largely dictates the immune profile at the tumor site; local production of numerous inflammatory mediators is crucial for the recruitment and activation of leukocytes in addition to macrophages and mast cells [48,63]. In particular, CD8 T cells and some types of innate immune cells, such as NK cells, can protect against experimental tumor growth [48,64].

Thus, the present study showed that P-MAPA increased significantly TLR2, TLR4 and p53 protein levels. In addition, it was demonstrated that this immunomodulator was more effective in the treatment of BC compared to BCG. These results were correlated with the ability of P-MAPA to act as TLR ligand, mainly for TLR2 and TLR4. The increased TRL2 and TLR4 levels were fundamental for antitumor immunotherapy of BC.

Furthermore, the present study demonstrated that the MNU animal model could lead to BC, besides kidney failure and increased alkaline phosphatase activity. Proctor *et al.*[65] demonstrated that increased alkaline phosphatase activity was associated with the presence of cancer and could be associated with mortality.

This MNU-Citrate animal model has particular advantages for the experimental analysis of complete carcinogenesis, since the carcinogen can be administered directly in quantifiable pulse doses, via intravesical instillation [66,67]. In addition, this autochthonous BC model includes low cost, reproducibility and an immunocompetent host, which is important, for example, when studying intravesical BCG treatment of BC [26,67,68]. Bladders treated with intravesical MNU-Citrate develop progressive neoplastic changes and tumours become progressively less differentiated with time. These lesions progress from hyperplasia, atypia, carcinoma in situ (CIS) and papillary carcinoma to large bulky muscle invasive tumours that completely fill the bladder lumen, obstruct the ureters and kill the animal [26,67,69]. Furthermore, the MNU-Citrate animal model is similar to human urothelial carcinogenesis; it involves the effect of environmental agents (carcinogen, smoking) in a genetically susceptible substrate (Fisher 344 rats) [26]. Intravesical instillation of fractionated doses of carcinogen and promoter provided a more controlled cancer model than those using carcinogens in the diet or drinking water. Added to its effectiveness (100 % of induction), low cost (not dependent on high-cost technologies) and short period of induction, it is in a position of superiority considering other available models, being a useful model for further studies [26].

The ability of intravesically administered BCG to exert an antineoplastic effect on BC is widely accepted and serves as the basis for its clinical use [70,71]. This agent induces a complex systemic immune response comprising humoral and cellular components [72]. Recent studies have demonstrated that polymorph nuclear neutrophils (PMN) migrating to the bladder after BCG instillation release large amounts of TNF-related apoptosis-inducing ligand (TRAIL), along with cytokines that recruit other immune cells, suggesting that PMN play a key role in the antitumor response to BCG therapy [15,73]. BCG intravesical immunotherapy induces a well-described T-lymphocyte predominant inflammatory infiltrate in the bladder wall and induces cytokines in the bladder and in urine [74]. These cytokines have anti-tumor and anti-angiogenic activity, and they share common regulatory pathways [74]. A number of prior reports documented a direct antiproliferative effect of BCG on urothelial carcinoma cells [75]. Potential mechanisms contributing to this biological effect include cell cycle arrest and/or apoptosis. DiPaola & Lattime [71] demonstrated that BCG induced signaling alters the susceptibility of the cell to an apoptotic insult. Thus, the consensus is that BCG serves as an immune potentiator of lymphocytes, namely an adjuvant, via the maturation of DC [22]. It appears that the effects in bladder cancer are local only. There is no protection against the development of tumors in areas where there is no BCG contact (e.g., the distal ureter and prostatic urethra).

BCG use is limited in BC by treatment failure, adverse effects and intolerance that occur in over two-thirds of all patients and consist largely of irritative voiding symptoms including haematuria, dysuria and urgency [19]. Even major complications and death related to treatment have been described [76]. In the BC studies presented here, the results demonstrated that P-MAPA immunotherapy was more effective in restoring normal morphological features and alkaline phosphatase activity compared to BCG. Concerning the toxicological analysis, the present results showed that both P-MAPA and BCG treatments did not show hepatotoxicity and nephrotoxicity compared to MNU group, which showed higher creatinine and urea serum levels; and indicating that high molecular weight of P-MAPA and BCG probably hinder local diffusion into the bladder wall thereby preventing systemic toxicity. Although BCG did not cause systemic toxicity, this immunotherapy led to intense local adverse effects such as haematuria compared to P-MAPA treatment, which was absent.

In addition, P-MAPA also increased the p53 protein level in the BC cancer model. The p53 gene and protein expression levels both play a critical role in the regulation of the normal cell cycle, cell cycle arrest, and apoptotic response [77-79]. Alterations in the p53 protein, leading to a loss of its tumor suppressor function, have been reported previously by some authors [79,80]. The p53 gene status has been examined in a number of malignancies, including cancers of bladder, breast, lung, ovary and colorectal cancer [79-83]. Studies of promoter response element sequences targeted by the p53 master regulatory transcription factor suggest a general role for this DNA damage and stress-responsive regulator in the control of human TLR gene expression [84]. Most of the TLR genes respond to p53 via canonical as well as noncanonical promoter binding sites [84]. Expression of all TLR genes, TLR1 to TLR10, in blood lymphocytes and alveolar macrophages from healthy volunteers can be induced by DNA metabolic stressors [84]. Also, Menendez *et al.*[84] demonstrated that all TLR genes showed responses to DNA damage, and most were p53-mediated.

In conclusion, the presented results showed that P-MAPA was able to improve, and/or re-establish the immunocompetence when the immune system was impaired, by cancer and possibly in infectious diseases, resulting in remarkable therapeutic effects. The P-MAPA therapy showed stimulatory effect on TLRs and p53 that correlated with the decrease of cancer state. Furthermore, P-MAPA induced significant responses in vivo against TB. Thus, these results pointed out that P-MAPA hypothetically acted in a common control mechanism for both infectious diseases and cancer, which involved TLRs and p53 signaling pathway. Finally, the low toxicity of P-MAPA combined with its significant antimycobacterial and antitumor activities warrant its development as a potential candidate for adjuvant treatment of cancer and infectious diseases.

### Perspectives

This work shows the toll-like stimulating properties of P-MAPA in vitro and in vivo. The immunomodulator P-MAPA alone showed significant efficacy against *Mycobacterium tuberculosis (Erdman strain)* in vivo when administered at 5 mg/kg. P-MAPA did not show any direct antibactericidal activity in vitro against *Mycobacterium tuberculosis* (H37Rv).Taken together data obtained suggest an immunotherapeutic effect of the compound against tuberculosis in vivo. Stimulation on TLRs is a feasible possibility to explain this effect, to be explored in additional studies.

Since the P-MAPA has not shown antagonic effects on the antimicrobial action of MFX in vivo, we aim to assay the compound together with other antitubercular compounds at lower doses of antimicrobials, or by shortening the period of time administration of such medicines. The low in vivo toxicity of P-MAPA is another benefit of the compound. Experiments on P-MAPA activity on Cytochrome P-450 are under way which may provide additional data on the potential for interaction with other compounds.

In a series of experiments evaluating the potential of P-MAPA in cancer models, the toll-like ligand properties of P-MAPA were tested in vitro and subsequently the use of the compound alone and compared with BCG in an animal model when the immune system is impaired by carcinogen and with BC.

The results indicate the stimulatory effect of P-MAPA and BCG on TLR2, TLR4 and p53 in vivo. More importantly, the results correlated with a re-establishment of immunocompetence and a significant therapeutic effect in the treated animals was seen for both compounds in relation to controls. Thus, looking ahead, the data provides instigating insights for a possible use of P-MAPA as adjuvant with BCG or other therapies aiming to boost its effects against cancer.

An important question to be answered concerns the determination of the optimal dose for the immunomodulator P-MAPA to be used alone or in combinations with other drugs. Like other biological therapies, the in vivo response to P-MAPA may be not linear and hence lower doses or single dose schedules may achieve improved responses in vivo. Previous results from experiments concerning the use of P-MAPA in the Punta toro virus (PTV) infected mice model suggests that single dose schedules may work better than repeated doses [25]. Further studies are being planned to elucidate this question using animal models for the study of infectious diseases and cancer.

The modification of antioxidant enzyme activity that plays an essential role in cellular defense mechanisms against oxygen toxicity has been observed in various cancers. The importance of antioxidants and reactive oxygen species (ROS) in carcinogenesis is considered in experimental studies in vivo. The Nuclear Factor-κB (NF-κB) signaling pathway plays an important role in inflammation, cancer and stress responses. Some NF-κB targets, such as the Cytochrome p450, CYP1B1, antioxidants and, mainly TLRs, have been implicated in modulating cellular redox potential. Thus, studies concerning the TLR and its signaling pathways associated with antioxidants and ROS are being planned to investigate the role of P-MAPA as an antioxidant agent in vitro and in vivo. These studies may provide useful data to complement the understanding of the P-MAPA´s mechanism of action.

## Methods

### In Vitro Assays

#### P-MAPA assay procedure: Human TLR Ligand Screening

The effect of P-MAPA on TLRs was performed through NF-κB activation in HEK293 cells expressing a given TLR (InvivoGen, San Diego, CA, USA). The secreted alkaline phosphatase (SEAP) reporter is under the control of a promoter inducible by the transcription factor NF-κB. This reporter gene allowed the monitoring of signaling through the TLR, based on the activation of NF-κB. The activity of P-MAPA (Farmabrasilis, Campinas, SP, Brazil) in a screening test was assessed in seven different human TLRs (TLR2, 3, 4, 5, 7, 8 and 9). The Control ligands for TLRs were: TLR2: HKLM (heat-killed *Listeria monocytogenes*) at 10^8^ cells/ml; TLR3: Poly(I:C) at 1 μg/mL; TLR4: *E. coli* K12 LPS at 100 ng/ml; TLR5: *S. typhimurium* flagellin at 100 ng/ml; TLR7: CL097 at 1 μg/mL; TLR8: CL075 at 1 μg/mL; TLR9: CpG ODN 2006 at 1 μg/mL; NF-κB Control cells: TNFα at 100 ng/ml (InvivoGen, San Diego, CA, USA). The P-MAPA (5 mg) was diluted with 1 mL of dimethyl sulfoxide (DMSO) for a stock concentration of 5 mg/mL. The P-MAPA samples were tested at final concentration of 50, 5 and 0.5 μg/mL and compared to control ligands. This step was performed in duplicate (InvivoGen, San Diego, CA, USA).

In a 96-well plate (200 μL total volume) containing the 50,000 cells/well, 20 μL of P-MAPA sample or the positive control ligands were added to the wells. The media was added to the wells, which was designed for the detection of NF-κB induced SEAP expression. After a 16–20 h incubation the optical density (OD) was read at 650 nm on a Beckman Coulter AD 340 C Absorbance Detector (InvivoGen, San Diego, CA, USA).

#### Resazurin MIC (Minimum Inhibitory Concentration) Assay and Single Point Concentration Procedure

The antimycobacterial activity of P-MAPA was tested at a concentration of 10 μg/ml against *Mycobacterium tuberculosis* H37Rv (H37Rv), obtained from Colorado State University, Fort Collins, CO (SRI International, Menlo Park, CA, USA). Upon receipt, test P-MAPA was logged into the inventory spreadsheet and placed in a −20 °C freezer [85]. On the day of the experiment, one vial from P-MAPA was reconstituted using DMSO to achieve a stock concentration of 3.2 mg/ml. Initially, P-MAPA compound was screened by means of in vitro tests (resazurin MIC assay) using a single concentration of 10 ug/ml of the compound (single point concentration procedure) against *M.tuberculosis* (H37Rv strain). Positive controls such as Isoniazid (INH) and Rifampin were used for comparative purposes. If compounds are active below 10 μg/ml, they are further tested in a MIC assay at 8 concentrations in a dose range between 10 ug/ml to 0.078 ug/ml. [85]. H37Rv was grown in Middlebrook 7 H9 broth medium (7 H9 medium) supplemented with 0.2 % (v/v) glycerol, 10 % (v/v) ADC (albumin, dextrose, catalase), and 0.05 % (v/v) Tween 80. The bacteria were inoculated in 50 ml of 7 H9 medium in 1 liter roller bottles that were placed on a roller bottle apparatus in an ambient 37 °C incubator. When the cells reach an OD600 of 0.150 (equivalent to ~1.5 x 10^7^ CFU/ml), they were diluted 200-fold in 7 H9 medium. After, 20 μl of the 3.2 mg/ml test P-MAPA were added to a 96-well microtiter plate. Also, 2-fold dilutions were made by the addition of 20 μl of diluent. Each dilution was further diluted 1:10 in sterile water (10 μl of dilution to 90 μl of sterile water). The additional 10-fold dilution in water was required when DMSO was used as solvent to minimize toxicity to the bacteria. Each dilution (6.25 μl) was transferred to duplicate 96-well test plates. After, 93.75 μl of the cell suspension (~ 10^4^ bacteria) in 7 H9 medium were added to the test plates (SRI International, Menlo Park, CA, USA).

Positive, negative, sterility and resazurin controls were tested. Positive controls included rifampicin and isoniazid; Negative controls included cell culture with solvent and water, or cell culture only; Sterility controls included media only or media with solvent and water. Resazurin control included one plate containing the diluted P-MAPA with resazurin only (SRI International, Menlo Park, CA, USA).

The 96 well test plates were incubated in an ambient 37 °C incubator for 6 days. After the 6 days of incubation, 5 μl of a 0.05 % sterile resazurin solution were added to each well of the 96-well plate. The plates were placed in an ambient 37 °C incubator for 2 days. After the 2 day incubation, visual evaluation and fluorimetric read-out were performed. The results were expressed as μg/ml (visual evaluation) and as IC_50_ (fluorimetric read-out). The single point concentration procedure was the same as the one used for the above described MIC procedure, but only a two-fold dilution was made in order to reduce the stock concentration to 1.6 mg/ml. An additional 1:10 dilution was made in water which further reduces the stock solution to 0.16 mg/mL. Addition of 6.25 μl of the 1:10 dilution to the wells in a final volume of 100 μl will give rise to a concentration equivalent to 10 μg/ml.

Only compounds that showed activity less than 10 μg/ml concentration were submitted to fluorimetric read-out analysis.

### In Vivo Assays

#### P-MAPA and Moxifloxacin (MXF) Activities against Mycobacterium tuberculosis (Erdman Strain)

Female C57BL/6 mice, 6 and 8 weeks old, were obtained from the Charles River Laboratories (Wilmington, MA). These animals were housed up to 5 animals per cage in HEPA-filtered racks (Thoren Caging Systems Inc. Hazleton, PA) in certified animal biosafety level 3 (ABSL-3) laboratories for at least 2 weeks before infection with *Mycobacterium tuberculosis*.

The *Mycobacterium tuberculosis* strain used was Erdman (TMC 107, ATCC 35801). The strain was grown initially as a pellicle as described previously by North & Izzo [86] with the following modifications. Briefly, the original vial was resuspended in Proskauer-Beck (PB) media and cultured as a pellicle for three passages. The final pellicle was harvested, disbursed into PB media with Tween 80 (Sigma Chemical Co., St Louis, MO, USA), and snap frozen after addition of glycerol (20 % total) as 1 ml seed stock cultures. Working stocks (maximum of 3 passages) were expanded into either PB (for infectivity stocks) or glycerol alanine salts (GAS) (for large scale growth stocks) media to mid-log phase from the seed stocks. Infectivity stock vials are recovered from frozen storage prior to use in animal model studies.

P-MAPA was provided by Farmabrasilis (Farmabrasilis fermentation pilot plant, Brazil) and 5 mg/kg of the compound were administered via intraperitoneal injection in 1 X PBS (3 x per week). Moxifloxacin (MXF) was administered daily at 50 mg/kg, by oral gavage, 5 days per week (at 0.2 ml per mouse) [87]. MXF was prepared in water. Organ harvesting was done at least 48 hours after last drug administration for the group to allow for clearance of the drugs prior to harvest of organs.

Written consent was obtained for all animal studies after institutional review and approval by the Animal Care and Use Committee at the Colorado State University. Six- to 8-week-old female specific pathogen-free immunocompetent C57BL/6 mice (Charles River, Wilmington, MA) were infected via a low dose aerosol exposure to *Mycobacterium tuberculosis* Erdman in the Animal Biosafety Level-3 Laboratories (ABSL3), as described by Kelly et al. [88]. Three mice were euthanized one day post low dose aerosol to verify bacterial uptake of 50 to 100 CFU per mouse. Treatment was started 3 weeks post-infection and continued for 4 weeks. Five infected mice were euthanized at the start of treatment as pretreatment controls. Mice were euthanized by CO_2_ inhalation 4 weeks after the start of treatment.

For enumeration of bacterial colonies on culture plates, the left lung lobes were aseptically removed and disrupted in a tissue homogenizer as previously described by Lenaerts et al. [87]. The number of viable organisms was determined by plating serial dilutions of the homogenates on nutrient Middlebrook 7 H11 agar plates (GIBCO BRL, Gaithersburg, MD). The plates were incubated at 37 °C in ambient air for 4 weeks prior to the counting of viable *Mycobacterium tuberculosis* colony forming units (CFU).

The antimycobacterial activity analyses were expressed as the log10 (and as log10 reduction) provided by a given dose of the MXF, P-MAPA and MXF + P-MAPA against the growth of the *Mycobacterium tuberculosis* in the untreated control group. Compounds with log10 protection factors > 0.60 were considered active [87].

#### Experimental design in Bladder Cancer

Sixty 7-week-old female Fisher 344 rats were obtained from the Multidisciplinary Center for Biological Investigation (CEMIB) at University of Campinas (UNICAMP). Forty-five animals were anesthetized with 10 % ketamine (60 mg/kg, i.m.; Vibra® Roseira, São Paulo, Brazil) and 2 % xylazine (5 mg/kg, i.m.; Vibra® Roseira, São Paulo, Brazil) and received 1.5 mg/Kg dose of *n-methyl-n-nitrosourea* (MNU) dissolved in 0.30 mL of sodium citrate (1 M pH 6.0); through a 22-gauge angiocatheter intravesically every other week for 8 weeks [26] The animals remained anesthetized for approximately 45 minutes after catheterization to prevent spontaneous micturition. The other 15 animals (Control group) received 0.30 ml dose of 0.9 % physiological saline, intravesically every other week for 7 weeks After MNU treatment, the 60 rats were divided into 4 groups (15 animals per group): The Control (CT) group received 0.30 ml dose of 0.9 % physiological saline intravesically every other week for 8 weeks; The MNU group (Bladder Cancer) received the same treatment as the CT group; The BCG group received 10^6^ CFU (40 mg) dose of BCG intravesically every other week for 8 weeks; The P-MAPA group received 5 mg/kg dose of P-MAPA (Farmabrasilis, Campinas, SP, Brazil) intravesically every other week for 8 weeks.

After 16 weeks of treatment, all animals were submitted to cystography to evaluate the occurrence of tumor. After that, the rats were euthanized and urinary bladder was collected, processed for histopathological and Western Blotting analysis. All protocols involving animal care and use were approved by the Institutional Committee for Ethics in Animal Experimentation (CEUA/IB/UNICAMP – 2684–1).

For histopathological analysis, urinary bladders were randomly collected from 10 animals in each group, fixed by immersion in 4 % paraformaldehyde, embedded in paraffin, cut into 5-μm thick and stained with hematoxylin-eosin. The neoplastic lesions were diagnosed using the nomenclature proposed by the World Health Organization/International Society of Urological Pathology consensus classification [89].

#### Western Blotting Analysis of TLR2, TLR4 and p53

Urinary bladders were randomly collected from 5 animals in each group, frozen in liquid nitrogen, weighed and homogenized in 50 μl/mg of lysis buffer. The tissue homogenates were centrifuged and a sample of each extract was used for protein quantification by Bradford`s method. Aliquots containing 50–70 μg of protein were separated by SDS-PAGE on 12 % polyacrylamide gels under reducing conditions. After electrophoresis, the proteins were transferred to Hybond-ECL nitrocellulose membranes (Amersham, Pharmacia Biotech, Arlington Heights, IL., USA). The membranes were blocked with TBS-T containing 1 % BSA (bovine serum albumin) and incubated at 4 °C overnight with primary rabbit polyclonal antibody ab13855 (abcam, USA) for the TLR2, mouse monoclonal ab30667 (abcam, USA) for the TLR4 and mouse monoclonal ab26 (abcam, USA) for the p53 (diluted 1:2,000; 1:2,000 and 1:1,500, respectively in 1 % BSA). The membranes were then incubated for 2 h with rabbit or mouse secondary HRP-conjugated antibodies (diluted 1:3,000 in 1 % BSA; Santa Cruz Biotechnology, Santa Cruz, CA, USA). Peroxidase activity was detected with SuperSignal West Pico Chemiluminescent Substrate kit (Pierce Biotechnology, Rockford, Illinois, USA). The luminescent signal from Western blot bands was captured by a G:Box iChemi camera (Syngene, Cambridge, UK) and band intensities were quantified using the analysis software provided by the manufacturer (Gene Tools Version 4.01, Syngene, Cambridge, UK). The results were expressed as means of ratio between each band intensity compared with β-actin band intensity.

#### Toxicological and Biochemical Analysis

In order to investigate possible hepatotoxic effects of P-MAPA and BCG the following serum analyses were carried out: activity of alanine aminotransferase (ALT), a specific marker for hepatic parenchymal injury; aspartate aminotransferase (AST), nonspecific markers for hepatic and/or cardiac injury; as well as the circulating levels of creatinine and urea in order to verify renal function. The alkaline phosphatase activity was verified to BC progression. The spectrophotometric determinations were performed in a Pharmacia Biotech spectrophotometer with temperature-controlled cuvette chamber (UV/visible Ultrospec 5,000 with Swift II applications software to computer control, 97–4213, Cambridge, England, UK). All chemicals were from LaborLab (Guarulhos, São Paulo, Brazil).

### Statistical Analysis

The Western Blotting, human TLR ligand screening, in vitro antimycobacterial activity and toxicological and biochemical analyses were statistically compared among the groups by analysis of variance followed by the Turkey’s test, with the level of significance set at 1 % and 5 %. The results were expressed as the mean ± standard deviation.

For in vivo antimycobacterial activity analyses, the CFU were converted to logarithms (log_10_CFU), which were then evaluated by multiple comparison analyses of variance including a one-way ANOVA test followed by a two-way ANOVA. For early treatment data, an ANOVA F-test was done to compare all treatments followed by comparisons of specific pairs of means. When the log counts were <2 logs and contained a large proportion of zeros, the Wilcoxon Rank Sum test was used.

## Abbreviations

ALT, Alanine Aminotransferase; AST, Aspartate Aminotransferase; BC, Bladder Cancer; BCG, Bacillus Calmette-Guerin; BSA, Bovine Serum Albumin; CIS, Carcinoma in situ; DC, Dendritic Cell; DMSO, Dimethyl Sulfoxide; IFN-γ, Interferon-gamma; IL, Interleukin; MIC, Minimum Inhibitory Concentration; MNU, n-methyl-n-nitrosourea; MXF, Moxifloxacin; NF-κB, Nuclear Factor-κB; NK, Natural Killer Cell; PAMP, Pathogen-Associated Molecular Patterns; P-MAPA, Protein Aggregate Magnesium-Ammonium Phospholinoleate-Palmitoleate Anhydride; PMN, Polymorph Nuclear Neutrophils; PTV, Punta Toro Virus; ROS, Reactive Oxygen Species; SEAP, Secreted Alkaline Phosphatase; TB, Tuberculosis; TLR, Toll-Like Receptor; TUR, Transurethral Resection; TRAIL, TNF-related Apoptosis-Inducing Ligand.

## Competing interests

The authors declare that they have no conflict of interest.

## Authors’ contributions

All authors contributed equally to this work. Also, all Authors read and approved the final manuscript.
